# Taurine is a potential therapy for rheumatoid arthritis via targeting FOXO3 through cellular senescence and autophagy

**DOI:** 10.1371/journal.pone.0318311

**Published:** 2025-04-16

**Authors:** Qingcong Zheng, Rongjie Lin, Zhechen Li, Qingzhu Zheng, Weihong Xu

**Affiliations:** 1 Department of Spinal Surgery, The First Affiliated Hospital of Fujian Medical University, Fuzhou, China; 2 Department of Orthopedic Surgery, Fujian Medical University Union Hospital, Fuzhou, China; 3 Department of Laboratory Medicine, Fujian Medical University Union Hospital, Fuzhou, China; Rutgers: Rutgers The State University of New Jersey, United States of America

## Abstract

**Background:**

Rheumatoid arthritis (RA) is a chronic inflammatory autoimmune disease closely related to aging with unclear pathogenic mechanisms. This study aims to identify the biomarkers in RA, aging and autophagy using bioinformatics and machine learning and explore the binding stability of taurine to target utilizing computer-aided drug design (CADD).

**Methods:**

We identified differentially expressed genes (DEGs) for RA, then crossed with gene libraries for aging and autophagy to identify common genes (Co-genes). We performed Gene Ontology (GO), Kyoto Encyclopedia of the Genome (KEGG), and ClueGO analysis for Co-genes. The Co-genes were subjected to support vector machine-recursive feature elimination (SVM-RFE), Degree, and Betweenness algorithms to get hub genes, then verified by an artificial neural network (ANN). After continuing to perform least absolute shrinkage and selection operator (LASSO) and weighted gene co-expression network analysis (WGCNA) on Co-genes, the results were crossed with hub genes to obtain genes, which were imported into various validation sets for receiver operating characteristics (ROC) to identify key genes. We analyzed the microRNA/TF network, enriched pathways, and immune cell infiltration for key genes. The binding stability of taurine with the target protein was verified by CADD. Finally, we used Western blot for in vitro experimental verification.

**Results:**

We obtained 74 Co-genes enriched in RA, cellular senescence, and regulation of programmed cell death. The model prediction of hub genes works well in ANN. The key genes (MMP9, CXCL10, IL15, FOXO3) were tested in ROC with excellent efficacy. In RA, FOXO3 expression was down-regulated while MMP9, CXCL10, and IL15 expression were upregulated, and FOXO3 was negatively correlated with MMP9, CXCL10, and IL15. Two miRNAs (hsa-mir-21-5p, hsa-mir-129-2-3p) and four TFs (CTCF, KLF, FOXC1, TP53) were associated with key genes. The immune cells positively correlated with MMP9, CXCL10, and IL15 expression and negatively correlated with FOXO3 expression were Plasma cells, CD8 T cells, memory-activated CD4 T cells, and follicular helper T cells, aggregating in RA. The binding stability of taurine with FOXO3 was verified by molecular docking and molecular dynamics simulation. In vitro experiments have indicated that taurine can upregulate the expression of FOXO3 and treat RA through the FOXO3-Parkin signaling pathway.

**Conclusions:**

MMP9, CXCL10, IL15, and FOXO3 are biomarkers of RA, cellular senescence, and autophagy. Taurine might be a promising drug against RA via targeting cellular senescence and autophagy through FOXO3.

## 1 Introduction

Rheumatoid arthritis is a classic chronic autoimmune disease with a 1% prevalence worldwide [1]. The global age-standardized prevalence rate (ASPR) of RA in 2019 was reported epidemiologically to be 224.25 (95% UI: 204.94–245.99), with a significant increase in the global ASPR of RA from 1990 to 2019 [2]. Another study reported that among RA patients, the rates peaked at 70–74 and 75–79 ages for women and men, respectively [3]. The most prominent feature of RA is persistent synovitis and systemic inflammation, which often manifests as symmetric joint pain, progressive bone and cartilage destruction, and joint deformity [4]. The fibroblast-like synoviocytes (FLS) of RA can highly express chemokines (CXCLs), interleukins (ILs), and matrix metalloproteinases (MMPs), which lead to the formation of pannus in the synovium [5]. Infiltration of immune cells also plays a crucial role in disease progression in RA [6].

Aging is closely associated with several chronic diseases; cellular senescence bridges their interconnections [7]. *Hayflick et al.* first proposed that cellular senescence is an irreversible growth arrest of cells after prolonged culture, and two features of cellular senescence are nonproliferation as well as the appearance of the senescence-associated secretory phenotype (SASP) [8]. SASP includes various extracellular regulators, including CXCLs, ILs, and MMPs, that can cause damage to neighboring cells and tissues [9]. *López-Otín et al.* proposed nine biological hallmarks of aging, which are: genomic instability (e.g., DNA damage), telomere attrition, epigenetic alterations, loss of proteostasis (e.g., impaired autophagy), deregulated nutrient sensing (e.g., downregulation of FOXO expression), mitochondrial dysfunction (e.g., oxidative stress and ROS production), cellular senescence (e.g., SASP), stem cell exhaustion, and altered intercellular communication (e.g., inflammaging) [10], and these features can be recognized in advance in RA patients [11].

Autophagy is an important intracellular self-digestive behavior that can transport abnormal proteins and damaged organelles to lysosomes for degradation [12]. Inhibition/impairment in autophagy will accelerate aging, and autophagy plays an important role in delaying aging and mitigating aging-related diseases [13]. One of the important pathogenic mechanisms of RA is the impairment/dysregulation of autophagy [14]. Thus, impaired autophagy may play a key role in RA and aging. The FOXO family of transcription factors in mammals includes four types: FOXO1/-3/-4/-6. The FOXO protein is characterized by a “forkhead” DNA binding domain containing the recognizable common sequence TGTTTAC [15]. FOXO proteins are masters of balance, which act as downstream effectors regulated by multiple pathways through post-translational modifications (PTM) and have important roles in aging, autophagy, and DNA damage repair [16]. FOXO3 is the most important member of the FOXO family and is closely related to autophagy and cellular senescence. Related studies have shown that FOXO3 is important in the pathogenesis of autoimmune diseases such as RA [17]. LPS stimulation causes downregulation of FOXO3 in FLS cells, while FOXO3 overexpression significantly attenuates FLS cell proliferation and the release of inflammatory factors (IL-6 and IL-1β) [18]. In addition, FOXO3 has been identified as the main gene for longevity in humans [19]. FOXO3 can translocate to the nucleus and upregulate multiple autophagy-related genes, thereby promoting cell protective autophagy [20]. Thus, FOXO3 may be a key biomarker for RA, cellular senescence and autophagy.

Taurine is a sulfur-containing β-amino acid with various effects, including antioxidant and anti-inflammatory properties [21]. Taurine supplementation in aging mice extends the median life span by 10–12%, and low taurine levels are associated with various age-related diseases in humans. Taurine inhibits cellular senescence and reduces inflammation, and its deficiency is a driver of aging [22]. Taurine can control the progression of RA through various mechanisms, such as inhibiting oxidative stress and reducing inflammation [23]. In addition, taurine can activate autophagy and play an anti-inflammatory role by inhibiting the AKT/mTOR axis [24], and taurine can also accelerate autophagy by enhancing TFEB nuclear translocation through ERK1/2 [25]. There are various bioinformatics and machine learning methods. We use multiple methods to cross-validate each other to screen for disease targets of RA. The aim is to use the advantages of different tools in a complementary way to improve the accuracy and comprehensiveness of the analysis. We use computer-aided drug design to study the binding ability of taurine to targets, and finally verify the results through in vitro experiments to provide a theoretical basis for basic research, drug re-use and clinical diagnosis and treatment.

## 2 Methodology

### 2.1 Data collection and processing

Three datasets for RA were included: GSE55235 [26], GSE77298 [27], and GSE1919 [28], which were screened using the National Center for Biotechnology Information (NCBI) Gene Expression Omnibus (GEO) (https://www.ncbi.nlm.nih.gov/geo/) (Table 1). The GSE55235 dataset contains synovial tissue samples from 10 patients with rheumatoid arthritis and 10 normal subjects for deg identification. This dataset has a wide range of research applications and high quality, so it is a very representative test dataset. The GSE55235 dataset can provide a wealth of gene expression information to help reveal the underlying molecular mechanisms of RA, and is the core basic dataset in this study. The GSE77298 dataset contains synovial tissue samples from 16 patients with RA and 7 normal. The GSE1919 dataset contains synovial tissue samples from 5 patients with RA and 5 normal. The GSE77298 and GSE1919 datasets were derived from different studies and used different gene chip platforms. This provides validation of cross-study and cross-platform contexts for this study, enhancing the reliability and external consistency of the research conclusions, especially the validation of results in the context of different clinical samples. These three datasets do not provide complete information such as patient gender, age, and race, which prevents us from fully understanding the heterogeneity of the sample. Nevertheless, these dataset remains highly relevant and has an important validation role in our research design. In addition, we further verified the results through in vitro experiments. In summary, the selection of these three datasets allows us to explore the genetic characteristics of RA from multiple dimensions and comprehensively analyze gene expression patterns. We downloaded 278 cellular senescence-related genes from the Database of Cell Senescence Genes (CellAge) (https://genomics.senescence.info/cells/) [29] and 125 cellular senescence-related genes (SenMayo) from a previous report [30]. We pooled the above data and removed duplicates to obtain 390 CellAge-related genes. Finally, we downloaded 10,392 autophagy-related genes from the GeneCards database (https://www.genecards.org/) [31].

**Table 1:** Basic information of selected datasets.

Dataset ID	Platform	Tissue(Homo sapiens)	Rheumatoid arthritis	Normal control	Attribute
GSE55235	GPL96	Synovium	10	10	Test set
GSE77298	GPL570	Synovium	16	7	Validation set
GSE1919	GPL91	Synovium	5	5	Validation set

### 2.2 Identification of Co-genes

Limma (http://www.bioconductor.org/packages/release/bioc/html/limma.html) is a differential gene screening method based on generalized linear models. We normalized the GSE55235, GSE77298, and GSE1919 datasets and subsequently used the “limma” package in R software (|log2 FC | >  0.5 and *P* <  0.05 as the cutoff) to obtain RA-DEGs. We used the “ggplot2” package to plot the volcano map. For DEGs, aging and autophagy-related genes using the “VennDiagram” package were obtained Co-genes. We used a clustered heat map to present the up and down-regulated genes of Co-genes in GSE55235. Gene clustering is an important step in performing gene analysis. We further used the “stats” package, “umap” package, and “rtsne” package of unsupervised clustering methods for principal component analysis (PCA), uniform manifold approximation and projection (UMAP), and t-distributed stochastic neighbor embedding (t-SNE).

### 2.3 GO, KEGG, and ClueGO enrichment analyses

We used Go and KEGG analyses to explore the pathways and functions of Co-genes. The GO analysis was run in R software using the “org.Hs.e.g.,db” package and the “clusterProfiler” package, and the KEGG analysis was run using the “KEGG rest AP” package, and the “clusterProfiler” package. ClueGO is a kappa method to visualize target genes, and we put Co-genes through ClueGO to construct interactive gene network maps and analyze potential functions.

### 2.4 Protein-protein interaction (PPI) network, k-means clustering, and CytoHubba analysis

We used the STRING database (https://string-db.org/) to construct a PPI network for Co-genes with a confidence score >  0.40 as the cutoff. The k-means clustering algorithm is an unsupervised machine learning algorithm that efficiently predicts pairs of interacting proteins, and we set the number of clusters at 3 for clustering analysis of Co-genes. The PPI network and K-means clustering analysis data were imported into Cytoscape 3.9.1 software in TSV format for visualization. We analyzed gene clusters for k-means using the Degree algorithm, and Co-genes were processed using both Degree and Betweenness algorithms in CytoHubba.

### 2.5 SVM-RFE and enrichment analyses

By eliminating unimportant features through recursion, SVM-RFE can help us screen the most predictive genes from complex genetic data, ensuring the accuracy of the initial screening. SVM-RFE is an effective machine learning algorithm for discovering genomes with maximum discriminatory power [32], and we used the “e1071” package for Co-genes to pre-screen candidate hub genes. Metascape (https://metascape.org/gp/index.html#/main/step1) is a gene function analysis platform that combines more than 40 databases. We set *P* <  0.01, a minimum count of 3, and an enrichment factor >  1.5 to distinguish gene clusters and perform functional enrichment, and subsequently imported gene clusters with similarity >  0.3 into Cytoscape software for potential relationships between genes.

### 2.6 Degree, Betweenness algorithms, and ANN analyses

We want to obtain a more focused set of target genes through various algorithms. In Co-genes, the candidate hub genes obtained from Degree and Betweenness algorithms were crossed with those obtained from SVM-RFE and set as Model 1 and Model 2, respectively, which were further verified by ANN. ANN can capture the non-linear relationships in data and help us identify key genes that may be overlooked in high-dimensional data. Its powerful fitting ability makes it an effective tool for screening potentially important genes. In the ANN model, the gene data of Model 1 and Model 2 were normalized and filtered into “gene scores” using the min-max normalization method, and then the feed-forward neural network was constructed by running the “NeuralNetTools” and “neuralnet” packages (the random seed size was set at 12,345,678) [33]. The ANN diagnostic model consists of an input layer (gene scores), a hidden layer (creating 5 hidden nodes and using modified linear units as activation functions), and an output layer (2 nodes: HC and RA).

### 2.7 LASSO and WGCNA

LASSO is a machine learning algorithm for identifying feature genes [34], and we used the “glmnet” package for Co-genes to perform regression analysis and filtered by constraining the regression coefficients (λ) to obtain the optimal model. LASSO controls the selection of features through regularization methods, reduces the interference of redundant genes, and improves the prediction accuracy of the model. In our analysis, LASSO helped us further optimize the candidate gene set and ensure that the genes finally selected were biologically more relevant. WGCNA can be used to find modules of highly related genes to further screen hub genes [35]. By constructing gene co-expression networks, WGCNA reveals the synergies between genes and helps us identify gene modules related to research objectives. This method is particularly important for revealing complex interrelationships between genes. We used the “WGCNA” package for Co-genes to construct a scale-free co-expression network, employing Pearson’s correlation matrices and the average linkage method to calculate linear correlations between genes (categorizing genes with similar patterns into the same module and preserving the feature vectors to correlate with the module genes).

### 2.8 Validation of key genes

We used GSE77298 and GSE1919 as external validation sets to identify key genes and presented differential expression of key genes in RA and HC groups by boxplot. The three RA datasets used the “ pROC “ package to draw ROC curves and validate the classification efficiency by the area under the curve (AUC). In GSE55235, joy plot and violin plot were used to plot the expression distribution and expression amount of key genes. Finally, the linear correlation between key genes was plotted by the person correlation coefficient.

### 2.9 In vitro experimental validation

This study used human rheumatoid arthritis fibroblast-like synoviocytes (iCell Bioscience Inc, iCell-008a) for in vitro experimental verification. The priMed-iCell-003 was used as a culture medium for FLS. We placed the culture bottle in a 37°C, 5% CO_2_ incubator for static culture, changing the liquid every 2 days, until the cells reached the desired adherent growth state. After adjusting the cell density, FLS was inoculated in a 6-well cell culture plate and incubated in an incubator for 24 h. The control group was cultured in complete medium; the RA group was incubated with 100 ng/ml TNF-α (MCE, HY-P1860) for 24 h; the treatment group was incubated with 100 ng/ml TNF-α for 24 h, and then 150 μM taurine (MCE, HY-B0351) was added and incubated for another 24 h.

### 2.10 Western blot analysis

The protein concentration was quantified after extraction from FLS cells in each group. Each protein sample was separated by SDS-PAGE and then transferred to a PVDF membrane. After the transfer is complete, seal the film with 5% skim milk at room temperature for 2 h. Next, the PVDF membrane was incubated with the primary antibody to β-actin, FOXO3, Parkin and LC3B (1:1,000, MCE) overnight at 4°C. The next day, after washing the membrane with TBST, incubate with the secondary antibody at room temperature for 1 h. Finally, after the antibodies were colored by HRP labeling using the ECL kit, the strips were detected using a gel imaging system. The gray value of the band was analyzed using ImageJ software, and the relative content of the target protein was calculated based on β-actin. Three independent analyses were performed.

### 2.11 Construction of microRNA/TF-key genes regulatory network

Screening for microRNA and TF associated with key genes using NetworkAnalyst (https://www.networkanalyst.ca/). The miRTarBase v8.0 and TarBase v8.0 databases were used for screening microRNA, and the ENCODE and JASPAR databases were used for screening TF. Finally, microRNA and TF were visualized using Cytoscape, and the first-order network and minimum network were plotted.

### 2.12 Pathway enrichment analysis of key genes

GeneMANIA (http://www.genemania.org) integrates GEO, BioGRID, and Pathway Commons databases to predict the interactions between key genes and the 20 most relevant genes, which allows us to prioritize genes further and find corresponding functional pathways. SPEED2 (https://speed2.sys-bio.net/) was used to explore the upstream signaling pathways of key genes. The data were obtained from human cell biology-related experiments, and the correlation between the expression of key genes and 16 pathways can be predicted based on the *P*-value.

### 2.13 Gene Set Enrichment Analysis (GSEA)

We use GSEA in the Molecular Signature database to interpret shared pathways associated with expression data for key genes. We obtained the GSEA software (version 3.0) from the GSEA (http://software.broadinstitute.org/gsea/index.jsp). The samples were divided into a high-expression group and a low-expression group according to the expression level of each key gene in GSE55235. We downloaded the c2.cp.wikipathways.v7.4.symbols.gmt collection from the Molecular Signatures database (http://www.gsea-msigdb.org/gsea/downloads.jsp) to evaluate the enriched pathways [36].

### 2.14 Analysis of immune cell infiltration

CIBERSORT (http://CIBERSORT.stanford.edu/) is a linear support vector regression-based analysis capable of characterizing the infiltration and composition of immune cells in a dataset by RNA mixtures of biomarkers. We revealed the correlation between key genes and the abundance of immune cell subtypes by person correlation coefficient analysis.

### 2.15 Molecular docking

We downloaded the crystal structure of taurine from the PubChem database (https://pubchem.ncbi.nlm.nih.gov/) as the docking ligand and the crystal structure of the target protein from the PDB database (https://www.rcsb.org/) as the docking acceptor. We used the Maestro program in the Schrödinger software for molecular docking. The 2D structure of taurine (sdf format) was processed using the LigPrep module, and 3D conformations were generated. The target protein structure was prepared prior to molecular docking using the Protein Preparation Wizard module. The SiteMap module predicted the optimal binding sites for ligands and receptors. The Receptor Grid Generation module (by setting the most suitable enclosing box) was used to accurately wrap the predicted binding sites to obtain the target protein’s active site. Among HTVS, SP, and XP modes, XP (flexible docking) has the highest accuracy and reliability. We performed molecular docking of ligands and receptors using XP mode and obtained docking scores. Finally, the molecular mechanics generalized Born surface area (MM-GBSA) calculations were performed on the complexes to obtain binding free energy scores.

### 2.16 Molecular dynamics simulation (MDS)

We used the Desmond program in the Schrödinger software to complete the MDS. The ligand and receptor were parameterized using the OPLS4 force field, and the aqueous solvent was parameterized using the SPCE model. The taurine-target protein was placed in an SPC solvent box to constitute a complex-solvent system, and 0.150 m Na^ + ^/Cl^-^ was added to the system to equilibrate the system charge. We used the Molecular Dynamics module for formal simulations. We set the simulation temperature to 300 K, the pressure to 1.01325 bar, and performed a 100 ns NPT simulation after completing the two equilibrium phases. Finally, ligand-receptor interactions were analyzed using Maestro 2023, generating dynamic trajectory animations. The root mean square deviation (RMSD) and the root mean square fluctuation (RMSF) were analyzed throughout the simulation using the Simulation Interaction Diagram tool.

**Fig 1:**
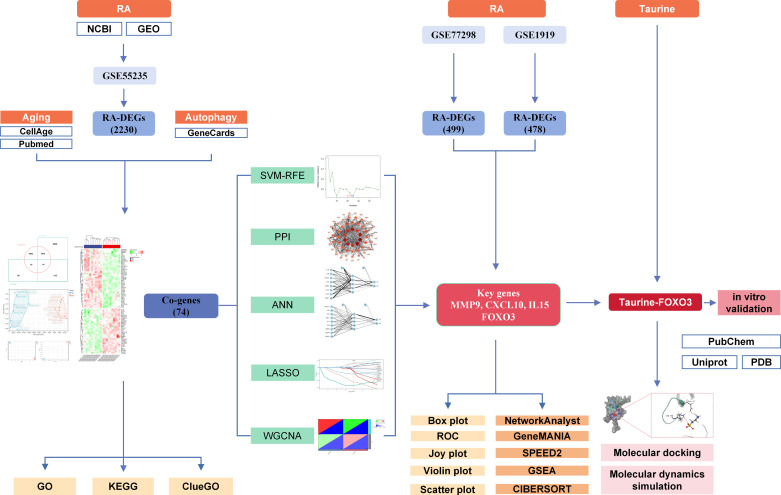
Flowsheet summarizing analysis strategy. RA, rheumatoid arthritis; DEGs: differentially expressed genes; GO, gene ontology; KEGG, Kyoto encyclopedia of genes and genomes; SVM-RFE: support vector machine-recursive feature elimination; PPI, protein-protein interaction; ANN: artificial neural network; LASSO: least absolute shrinkage and selection operator; WGCNA: weighted gene co-expression network analysis; ROC: receiver operating characteristic curve; GSEA: gene set enrichment analysis.

The flowchart of this study is shown in Fig 1, and all raw data has been uploaded to *GitHub (https://github.com/zheng5862/FOXO3.git)*.

## 3 Results

### 3.1 Processing of datasets and identification of Co-genes

**Fig 2:**
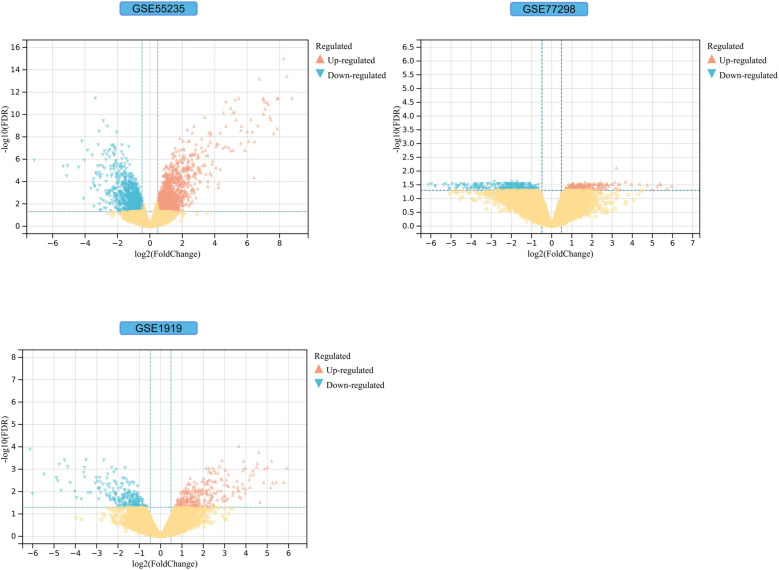
Identification of RA-DEGs. **Yellow squares indicate no significant difference in gene expression between the RA and HC groups** (*P* >  0.05); Blue and red triangles indicate significant differences in gene expression between the RA and HC groups (*P* <  0.05). Blue triangles identify genes down-regulated in RA, and red triangles identify genes upregulated in RA.

**Fig 3:**
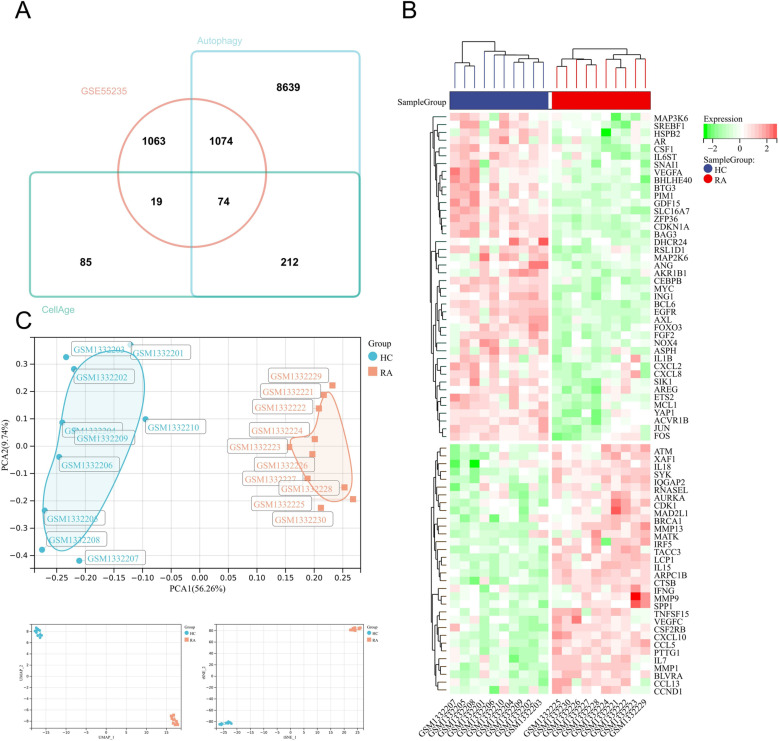
(A) Venn diagram of RA-DEGs, CellAge, and autophagy. (B) Heatmap of Co-genes in GSE55235. HC group in blue and RA group in red; the green color in the expression profile indicates gene down-regulation, and the red color indicates gene up-regulation. (C) PCA, UMAP, and t-SNE analysis of Co-genes in GSE55235. HC group in light blue and RA group in orange.

We obtained 2230, 499 and 478 DEGs from the GSE55235, GSE77298, and GSE1919 datasets by the limma (Fig 2). Co-genes included 74 genes (Fig 3A), of which 32 were upregulated, and 42 were down-regulated in GSE55235 (Fig 3B). PCA analysis of Co-genes in GSE55235 showed that PC1 was 56.26% and PC2 was 9.74%. UMAP and t-SNE plots showed significant categorization of RA and control groups. These three downscaling analyses exemplify Co-genes’ ability to significantly discriminate between RA and control with reliability (Fig 3C).

### 3.2 Functional enrichment analyses of Co-genes

**Fig 4:**
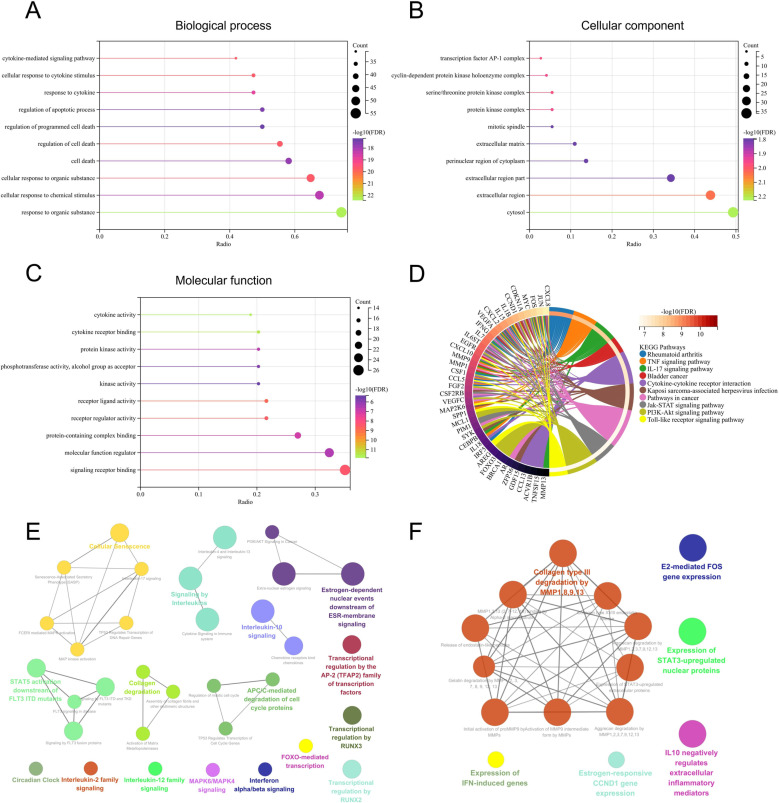
Functional enrichment analyses of Co-genes. (A) The biological process analysis of Co-genes. (B) The cellular component analysis of Co-genes. (C) The molecular function analysis of Co-genes. (D) KEGG analyses of Co-genes. (E) Reactome-pathways of Co-genes in ClueGo. (F) Reactome reactions of Co-genes in ClueGo.

In the biological process (BP), Co-genes were enriched in response to an organic substance, cell death, regulation of programmed cell death, response to cytokine, cellular response to cytokine stimulus, and cytokine-mediated signaling pathway (Fig 4A). In the cellular component (CC), Co-genes were enriched in the cytosol, extracellular matrix, and protein kinase complex (Fig 4B). In the molecular function (MF), Co-genes were enriched in signaling receptor binding, molecular function regulator, cytokine receptor binding, and cytokine activity (Fig 4C). In the KEGG, Co-genes were enriched in RA, TNF signaling pathway, IL-17 signaling pathway, cytokine-cytokine receptor interaction, and PI3K-AKT signaling pathway (Fig 4D). In the Reactome pathways of ClueGO, co-genes were enriched in cellular senescence, SASP, IL-17 signaling pathway, interleukins signaling pathway, FOXO-mediated transcription, and MAPK signaling pathway (Fig 4E). In the reactome-reactions of ClueGO, co-genes were enriched in collagen type III degradation by MMP1/-8/-9/-13, initial activation of pro-MMP9 by MMPs, activation of MMP9 intermediate form by MMPs (Fig 4F).

### 3.3 Screening of candidate hub genes and pathway enrichment analyses

**Fig 5:**
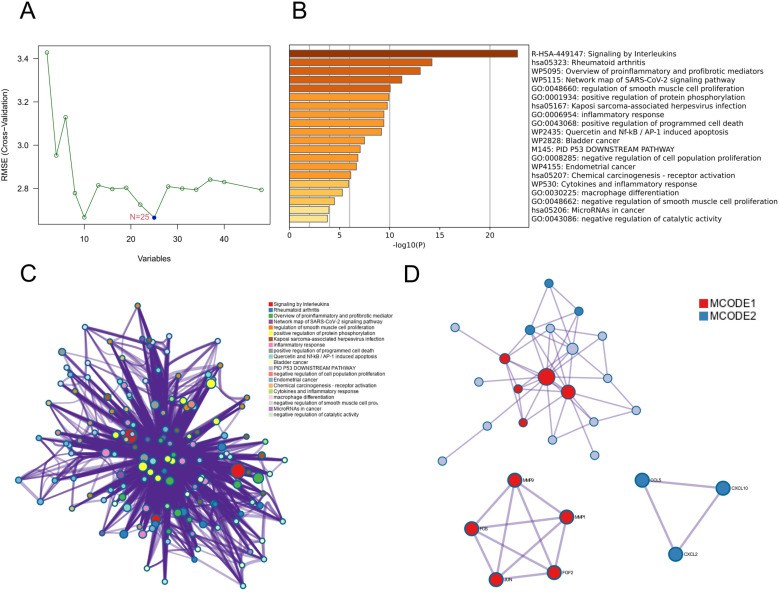
Candidate hub genes and enrichment analyses. (A) a plot of candidate hub genes screening via the SVM-RFE. (B) Bar graph of enriched terms. (C) Network of enriched terms. (D) PPI network and MCODE components identified candidate hub genes.

Twenty-five candidate hub genes (CCL5, ARPC1B, CXCL10, SYK, JUN, BTG3, FOS, MCL1, CXCL2, IL15, CTSB, GDF15, BCL6, ANG, SIK1, FGF2, BLVRA, IL1B, GRK6, MMP9, RSL1D1, CDN1A, VEGFA, FOXO3, and MMP1) were obtained from an initial screen of Co-genes using the SVM-RFE (Fig 5A). Candidate hub genes were enriched in RA, inflammatory response, interleukins signaling, cytokines and inflammatory response, and positive regulation of programmed cell death (Fig 5B). A network diagram was used to cluster and visualize the functional pathways (Fig 5C). The PPI enrichment analysis of candidate hub genes consisted of two modules: MCODE1 was enriched in the IL-17 signaling pathway, while MCODE2 was enriched in the chemokine-chemokine receptor pathway (Fig 5D).

### 3.4 Identification and validation of hub genes

**Fig 6:**
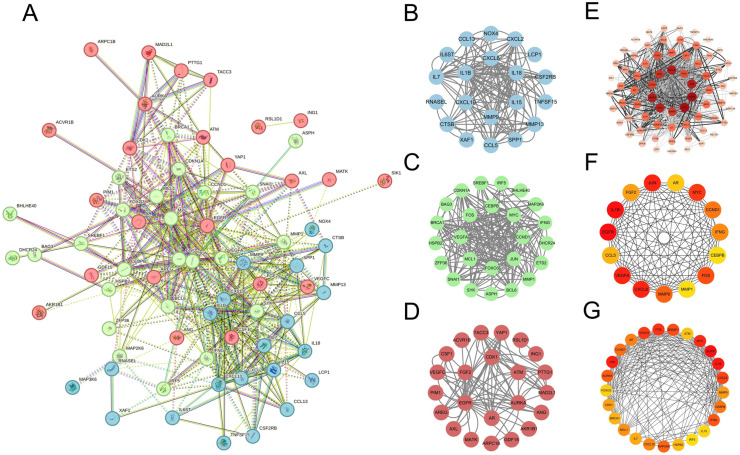
(A) PPI network of Co-genes. (B-D) Three blue, green, and red gene clusters were obtained using k-means for Co-genes. (E) Processing PPI data for Co-genes using Cytoscape. (F) Analyzing PPI data for Co-genes using the Degree algorithm. (G) Analyzing PPI data for Co-genes using the Betweenness algorithm.

**Fig 7:**
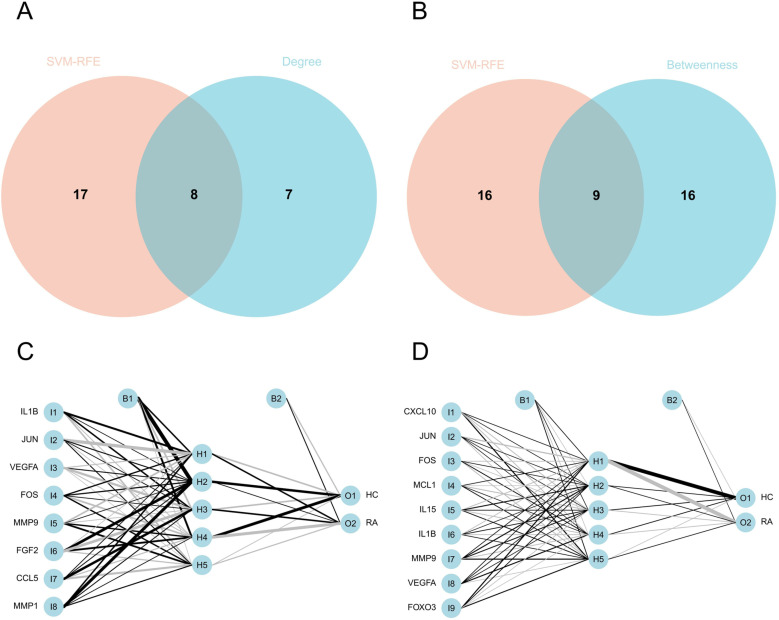
(A) Model 1: Crossover of genes screened by SVM-RFE and Degree algorithms. (B) Model 2: Crossover of genes screened by SVM-RFE and Betweenness algorithms. (C-D) Model 1 and Model 2 genes were validated by ANN.

The PPI network of Co-genes has 74 nodes and 492 edges (Fig 6A). By k-means clustering, the PPI data of Co-genes can be categorized into three gene clusters: blue, green, and red. Highly scored genes in the blue set were MMP9, CXCL10, IL15, IL1β, CXCL8, and IL18 (Fig 6B). Highly scored genes in the green set were FOXO3, MCL1, VEGFA, FOS, CEBPB, MYC, CCND1, and JUN (Fig 6C). Highly scored genes in the red set were FGF2, CDK1, ATM, AURKA, AR, and EGFR (Fig 6D). We imported the PPI data of Co-genes into Cytoscape and processed it to make the distribution of genes hierarchically obvious (Fig 6E). Highly scored genes obtained after using the Degree algorithm for Co-genes were EGFR, IL1β, CXCL8, JUN, VEGFA, MYC, FOS, MMP9, CCND1, IFNG, FGF2, CCL5, AR, MMP1, and CEBPB (Fig 6F). Highly scored genes obtained using the Betweenness algorithm for Co-genes were EGFR, IL1β, JUN, MYC, FOS, VEGFA, CXCL8, SREBF1, IFNG, AURKA, MAP2K6, CCND1, CEBPB, AR, CXCL10, MCL1, MMP9, CDK1, IL7, BRCA1, HSPB2, ATM, FOXO3, IL15, IRF5 (Fig 6G). Interestingly, the crossover of genes obtained by the Degree and Betweenness algorithms was essentially in the set of three highly rated genes of the k-means (except for IFNG). Finally, we obtained two sets of hub genes: the eight genes for Model 1 were IL1β, JUN, VEGFA, FOS, MMP9, FGF2, CCL5, MMP1 (Fig 7A), and the nine genes for Model 2 were CXCL10, JUN, FOS, MCL1, IL15, IL1β, MMP9, VEGFA, FOXO3 (Fig 7B). We used ANN for validation and found that both model tests were excellent (Fig 7C-D).

### 3.5 Identification of key genes

**Fig 8:**
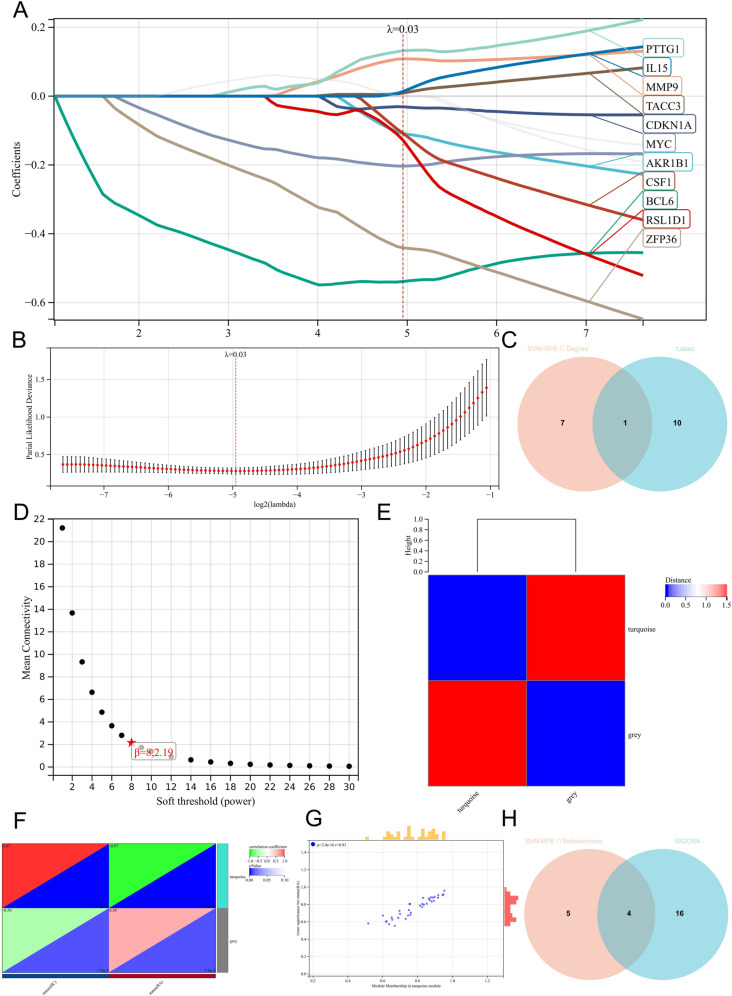
(A) LASSO regression analysis of Co-genes. (B) Cross-validation for adjusting the selection of parameters in LASSO. (C) Venn diagram of genes screened by SVM-RFE, Degree, and LASSO. (D) Analysis of the mean connectivity for soft threshold powers (β). (E) Modular eigenvector clustering of Co-genes. Blue represents a negative correlation, and red represents a positive correlation. (F) Heatmap of modular and phenotypic correlation of Co-genes. Green represents a negative correlation, and red represents a positive correlation. (G) Correlation of MM and GS in the turquoise module. (H) Venn diagram of genes screened by SVM-RFE, Betweenness, and WGCNA.

We obtained the coefficient relationship between the λ value and the independent variables in LASSO (Fig 8A) and the best fitting λ value of 0.03 in the cross-validation curve (Fig 8B). The 11 genes obtained at this λ value were AKR1B1, BCL6, CDKN1A, MMP9, MYC, PTTG1, RSL1D1, TACC3, ZFP36, CSF1, IL15. The key gene obtained by taking the intersection of the gene from Model 1 with the gene obtained from Lasso was MMP9 (Fig 8C). In WGCNA, mean connectivity reached a steady state at the optimal soft threshold β as 8 (Fig 8D). We obtained two gene modules, grey and turquoise (Fig 8E), and selected the turquoise module in the heat map of the correlation between modules and phenotypes (Fig 8F). In addition, Gene significance (GS) for status (RA) correlated well with Module membership (MM) in the turquoise module (Fig 8G). Under the condition of setting both MM threshold and GS threshold at 0.75, we obtained 20 genes were ARPC1B, BAG3, BCL6, CDKN1A, ETS2, FOXO3, MYC, SLC16A7, SYK, TACC3, ZFP36, CCL5, CXCL10, GDF15, IL15, IQGAP2, JUN, LCP1, MMP1, MMP13. The key genes obtained by taking the intersection of the gene from Model 2 with the gene obtained from WGCNA were CXCL10, JUN, IL15, and FOXO3 (Fig 8H).

### 3.6 Validation of key genes

**Fig 9:**
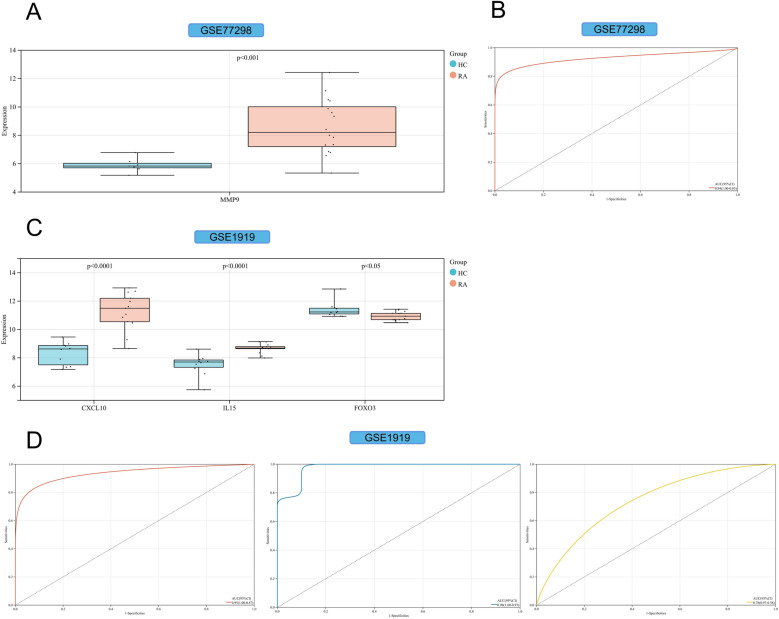
(A) Expression of MMP9 in GSE77298. (B) ROC curve of MMP9 in GSE77298. (C) Expression of CXCL10, IL15 and FOXO3 in GSE1919. (D) ROC curves of CXCL10, IL15, and FOXO3 in GSE1919. HC group in light blue and RA group in orange.

**Fig 10:**
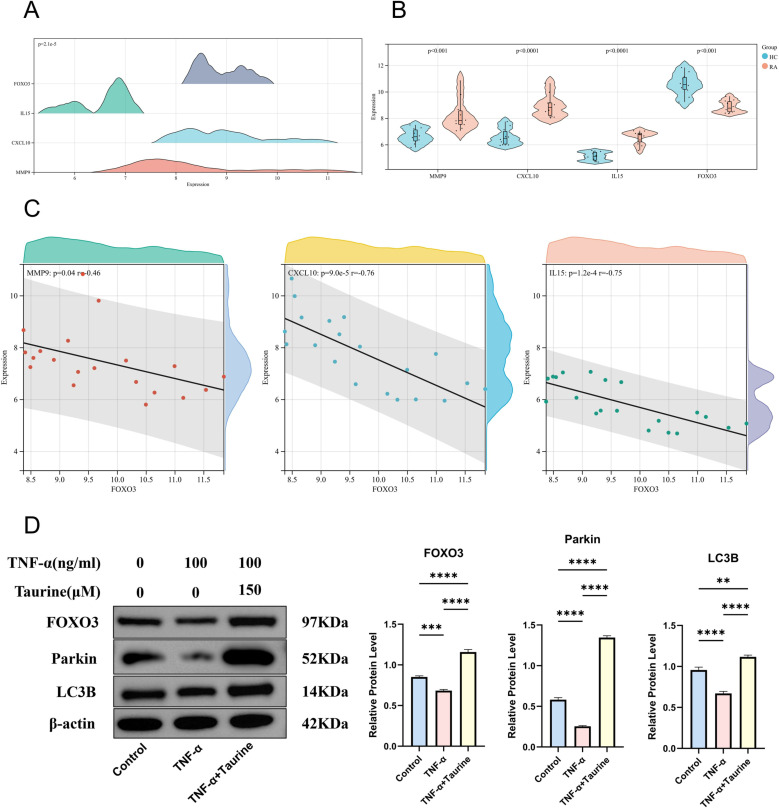
(A) Joy plot of key genes in GSE55235. (B) Violin plot of key genes in GSE55235. (C) Scatter plot of MMP9, CXCL10, and IL15 with FOXO3 in GSE55235, respectively. (D) In vitro experimental validation was performed using Western blot.

We obtained 499 DEGs (including 233 upregulated genes and 266 down-regulated genes) from GSE77298 and 478 DEGs (including 265 upregulated genes and 213 down-regulated genes) from GSE1919. In GSE77298, MMP9 was significantly higher expressed in the RA group than in the HC group (Fig 9A), and the AUC value of MMP9 in the ROC curve was 0.94 (Fig 9B). In GSE1919, CXCL10 and IL15 were significantly highly expressed, and FOXO3 was significantly less expressed in the RA group than the HC group (Fig 9C), and the AUC values of CXCL10, IL15, and FOXO3 in the ROC curves were 0.95, 0.98, and 0.78 (Fig 9D). In addition, JUN was excluded because there was no significant difference in the limma analysis in the RA and HC groups of the validation set. We provide strong support for the screening of key genes (MMP9, CXCL10, IL15 and FOXO3) through the effective combination of various bioinformatics and machine learning methods. We can view the expression distribution of key genes in GSE55235 (Fig 10A). MMP9, CXCL10, and IL15 were significantly highly expressed, and FOXO3 was significantly less expressed in the RA group compared with the HC group (Fig 10B). By constructing scatter plots of FOXO3 and the other three key genes, it can be found that FOXO3 is negatively correlated with MMP9 (*P* =  0.04, *R* =  -0.46), CXCL10 (*P* =  9.0e-5, *R* =  -0.76) and IL15 (*P* =  1.2e-4, *R* =  -0.75) (Fig 10C). To further confirm the expression status of FOXO3 in RA, whether the FOXO3-Parkin autophagy signal pathway is involved, and whether taurine is involved in the regulation of the FOXO3-Parkin pathway, we conducted in vitro experiments to verify these. The results showed that when FLS cells were in the disease environment of TNF-α-induced RA, the expression of FOXO3 decreased, and the levels of Parkin and LC3B autophagy-related proteins also decreased. Taurine can effectively inhibit the downregulation of FOXO3, promote the upregulation of Parkin and LC3B, and thus promote the autophagy process (Fig 10D).

### 3.7 MicroRNA/TF-key genes regulatory network

**Fig 11:**
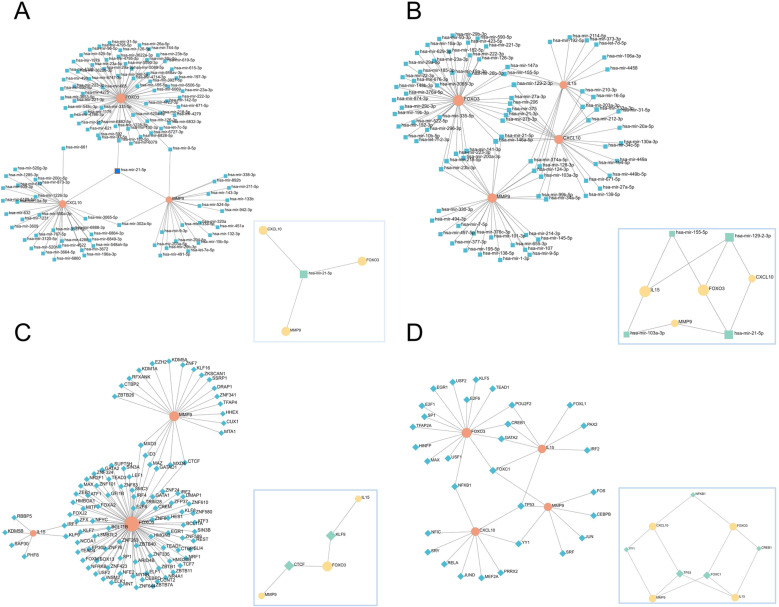
MicroRNA regulatory network of key genes. (A) miRTarBase databases. (B) TarBase databases. TF regulatory network of hub genes. (C) ENCODE databases. (D) JASPAR databases. The left side is the first-order network, and the bottom right corner is the minimum network.

The first-order network obtained from the miRTarBase database includes 115 nodes, 116 edges, and 3 seeds, and the minimum network includes 4 nodes, 3 edges, and 3 seeds (Fig 11A), from which the two plots show that hsa-mir-21-5p is closely related to MMP9, CXCL10, and FOXO3. The first-order network obtained from the TarBase database includes 90 nodes, 115 edges, and 4 seeds, and the minimum network includes 8 nodes, 10 edges, and 4 seeds (Fig 11B), from which the two plots show that hsa-mir-21-5p is closely associated with MMP9, CXCL10, and FOXO3, and hsa-mir-129-2-3p is closely associated with IL15, CXCL10, and FOXO3. The first-order network obtained from the ENCODE database includes 108 nodes, 113 edges, and 3 seeds, and the minimum network includes 5 nodes, 4 edges, and 3 seeds (Fig 11C), from which the two plots show that CTCF is closely related to MMP9, FOXO3, and KLF is closely related to IL15, FOXO3. The first-order network obtained in the JASPAR database includes 35 nodes, 40 edges, and 4 seeds, and the minimum network includes 9 nodes, 12 edges, and 4 seeds (Fig 11D), from which the two plots show that FOXC1 is associated with MMP9, IL15, and FOXO3, and TP53 is associated with MMP9, CXCL10, and IL15. Thus, two miRNAs (hsa-mir-21-5p, hsa-mir-129-2-3p) and four TFs (CTCF, KLF, FOXC1, and TP53) may play important roles in the expression of key genes.

### 3.8 Pathway enrichment analysis of key genes

**Fig 12:**
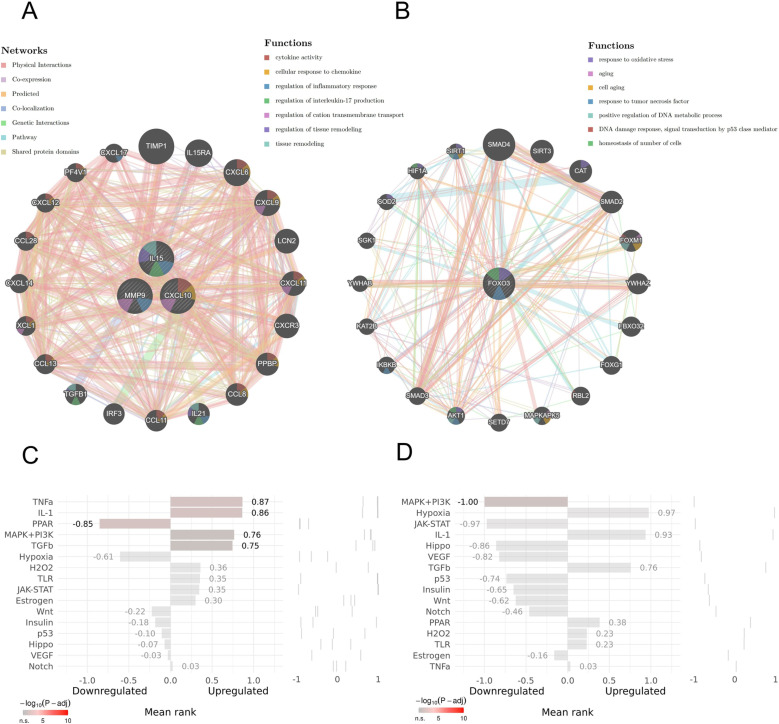
GeneMANIA analysis. (A) MMP9, CXCL10, and IL15. (B) FOXO3. SPEED2 analysis. (C) MMP9, CXCL10, and IL15. (D) FOXO3.

In GeneMANIA, MMP9, CXCL10, and IL15 were enriched with the 20 most relevant genes in cytokine activity, cellular response to chemokine, inflammatory response, regulation of IL-17 production, and tissue remodeling (Fig 12A). FOXO3 was enriched with the 20 most relevant genes in response to oxidative stress, aging, cell aging, DNA damage response, and cellular homeostasis (Fig 12B). In SPEED2, TNF-α, IL-1 and MAPK+PI3K signaling pathways positively regulate MMP9, CXCL10, and IL15 (Fig 12C), while MAPK+PI3K signaling pathways negatively regulate FOXO3 (Fig 12D).

### 3.9 GSEA of key genes

**Fig 13:**
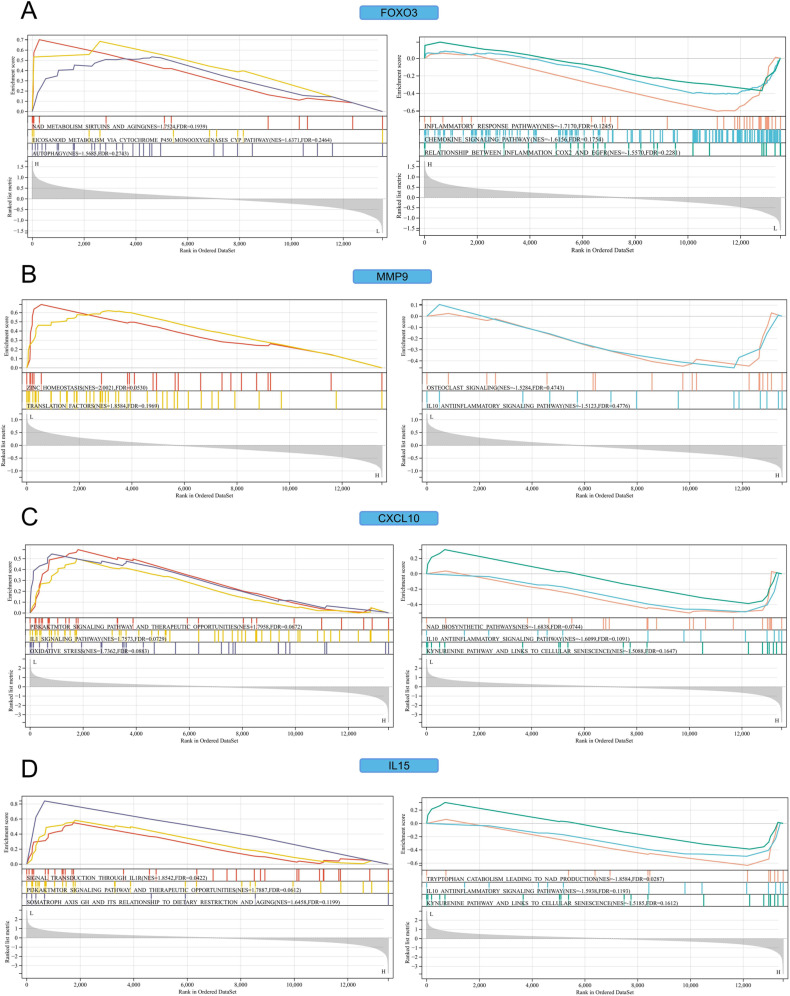
GSEA analysis. (A) FOXO3. (B) MMP9. (C) CXCL10. (D) IL15.

In Wikipathways gene sets, positively correlated with FOXO3 were NAD metabolism, sirtuins, and aging (*NES* =  1.7524, *FDR* =  0.1939), eicosanoid metabolism via cytochrome p450 monooxygenases CYP pathway (*NES* =  1.6371, *FDR* =  0.2464), and autophagy (*NES* =  1.5685, *FDR* =  0.2743). Negatively correlated with FOXO3 were the inflammatory response pathway (*NES* =  -1.7170, *FDR* =  0.1245), the chemokine signaling pathway (*NES* =  -1.6156, *FDR* =  0.1754), and the relationship between inflammation and COX2 and EGFR (*NES* =  -1.5570, *FDR* =  0.2281) (Fig 13A). Positively correlated with MMP9 were zinc homeostasis (*NES* =  2.0021, *FDR* =  0.0530) and translation factors (*NES* =  1.8584, *FDR* =  0.1969). The negatively correlated pathway with MMP9 was not significant (Fig 13B). Positively correlated with CXCL10 were the PI3K/AKT/MTOR signaling pathway and therapeutic opportunities (*NES* =  1.7958, *FDR* =  0.0672), the IL1 signaling pathway (*NES* =  1.7573, *FDR* =  0.0729), and oxidative stress (*NES* =  1.7362, *FDR* =  0.0883). Negatively correlated with CXCL10 were the NAD biosynthetic pathway (*NES* =  -1.6838, *FDR* =  0.0744), the IL-10 anti-inflammatory signaling pathway (*NES* =  -1.6099, *FDR* =  0.1091), the kynurenine pathway, and links to cellular senescence (*NES* =  -1.5088, *FDR* =  0.1647) (Fig 13C). Positively correlated with IL15 were signal transduction through IL1R (*NES* =  1.8542, *FDR* =  0.0422), the PI3K/AKT/MTOR signaling pathway, and therapeutic opportunities (*NES* =  1.7887, *FDR* =  0.0612), somatroph axis GH, and ITS relationship to dietary restriction and aging (*NES* =  1.6458, *FDR* =  0.1199). Negatively correlated with IL15 were tryptophan catabolism leading to NAD production (*NES* =  -1.8584, *FDR* =  0.0287), the IL-10 anti-inflammatory signaling pathway (*NES* =  -1.5938, *FDR* =  0.1193), the kynurenine pathway, and links to cellular senescence (*NES* =  -1.5185, *FDR* =  0.1612) (Fig 13D).

### 3.10 Immune infiltration analysis

**Fig 14:**
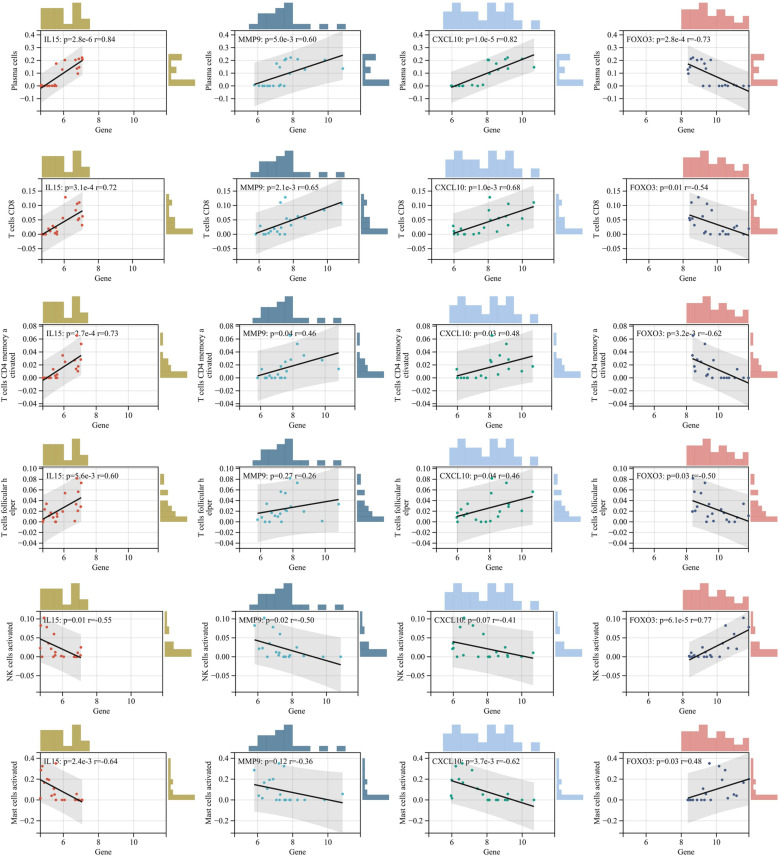
Correlation analysis of FOXO3 expression with immune cell infiltration.

In GSE55235, we obtained the correlation data of key genes with immune cell infiltration by CIBERSORT. Positively correlated with MMP9, CXCL10, and IL15 and negatively correlated with FOXO3: Plasma cells, CD8 T cells, memory-activated CD4 T cells, and follicular helper T cells, which accumulate in RA. Negatively correlated with MMP9, CXCL10, and IL15 and positively correlated with FOXO3: activated NK cells and activated mast cells (Fig 14).

### 3.11 Molecular docking

**Fig 15:**
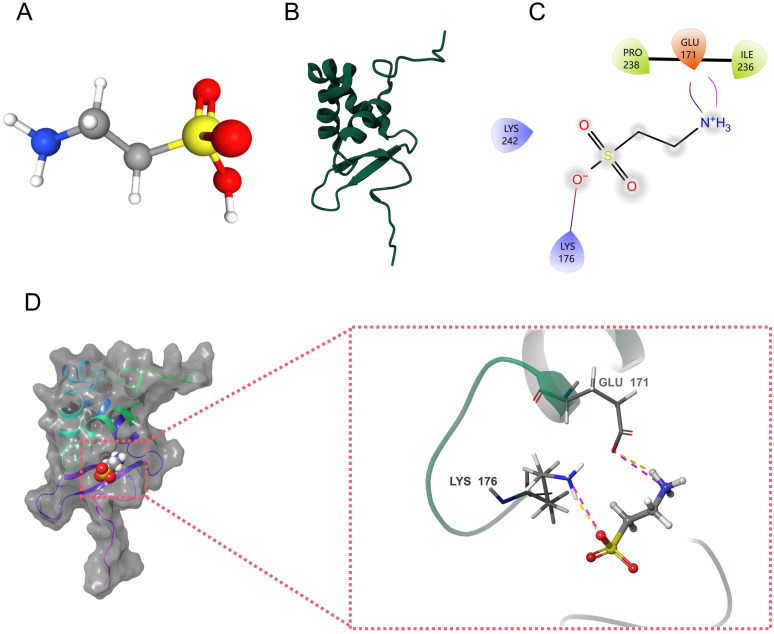
(A) 3D structure of the taurine molecule. (B) 3D structure of FOXO3 protein. (C) 2D plot of taurine docking with FOXO3 protein. (D) Binding mode of taurine and FOXO3 protein obtained based on molecular docking. The left figure shows the overall view and the right figure shows the partial view.

Molecular docking can predict the conformation of taurine within the target binding site of FOXO3 and assess the affinity for static binding of the ligand and receptor. We obtained the 3D crystal structure of taurine and the protein structure of FOXO3 (Fig 15A-B). As can be seen from the ligand-receptor interaction diagram, taurine binds to the active pocket of the FOXO3 protein. In Fig 15C-D, taurine forms ionic bonds with GLU171, hydrogen bonds with LYS176, and hydrophobic interactions with PRO238 and ILE236. The docking score of taurine to FOXO3 was -4.743 kcal/mol, and the MM-GBSA post-docking score was -8.88 kcal/mol, indicating that taurine has a high probability of binding to FOXO3.

### 3.12 Molecular dynamics simulation

**Fig 16:**
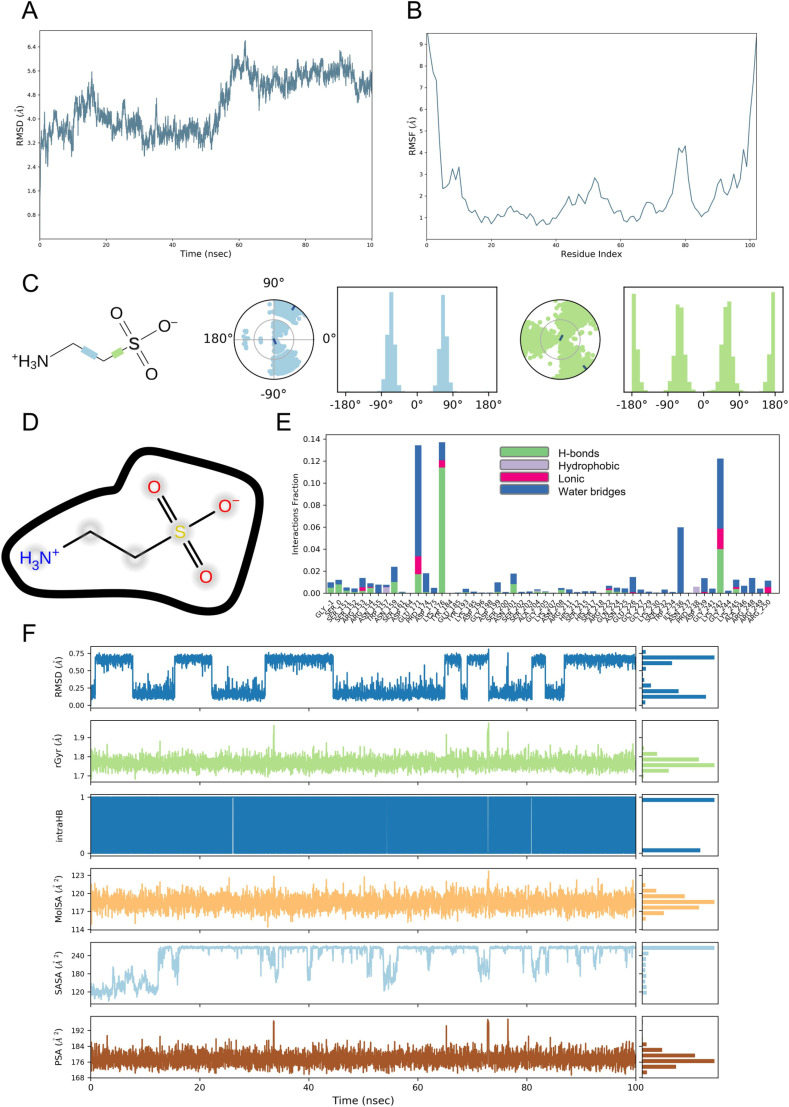
(A) RMSD of taurine-FOXO3 complex during the simulation. (B) RMSF of FOXO3 protein. (C) Conformational changes of rotatable bonds (RBs) in taurine throughout the simulation trajectory: The left side shows a 2D plot of taurine with blue and green RBs, and the right side plot shows a dial plot and bar chart of the RBs of the corresponding colors. (D) Schematic representation of the interaction of taurine with residues of FOXO3 protein. (E) Contribution of AAs at the binding site of FOXO3 and taurine to complex binding during MDS. Green is hydrogen bonds, purple is hydrophobicity, red is ionic bonds, and blue is water bridging. (F) RGyr, intraHB, MolSA, SASA, PSA of taurine-FOXO3 complex over time during MDS.

MDS enables further exploration of the dynamic binding properties of complexes and is a deepening experiment in molecular docking. We performed 100 ns MDS of taurine and FOXO3 proteins and analyzed the molecular dynamics trajectories of the complexes. Fig 16A shows the taurine-FOXO3 complex in RMSD, and the results indicated that the complex was relatively stable after 60 ns, and the system was in equilibrium. RMSF can be used to characterize local changes in protein chains, with peaks indicating protein regions that fluctuate the most during the simulation. Fig 16B shows that in taurine binding to FOXO3 protein, FOXO3 exhibits high structural flexibility in the residue regions of 0–10, 45–55, 75–85, and 90–100 amino acids (AAs). Fig 16C shows that the taurine can twist in both the blue and green positions, implying greater flexibility in that part. Fig 16D illustrates that taurine binding to FOXO3 relies primarily on solvent exposure. By MDS, we can observe that the main AAs that play an important role in the binding of taurine to the FOXO3 protein were GLU171, LYS176, ILE236, and LYS242, with the main forces being water bridges and hydrogen bonds (Fig 16E). Finally, we calculated the rGyr (radius of gyration), intraHB (intramolecular HBs), MolSA (molecular surface area), SASA (solvent accessible surface area), and PSA (polar surface area) of taurine over time using kinetic simulation trajectories. The results showed relatively stable fluctuations in these properties, suggesting that taurine can maintain basic physicochemical properties in the active binding pocket of the FOXO3 protein and undergo active action with consistent properties (Fig 16F).

## 4 Discussion

### 4.2 Relationship Between aging and RA

Important features of human aging include chronic and low-grade inflammation [37]. Senescent cells can autocrine and paracrine SASP to promote their accelerated senescence and the senescence of surrounding cells, and a positive feedback loop between aging and inflammation can be formed [38]. Indeed, whether it is aging or inflammation, oxidative stress and ROS accumulation are their common triggers [39]. **(1) Inflammaging.** Inflammaging is an inducer of unhealthy aging [40]. The FLS of RA can secrete large amounts of MMPs, CXCLs, and ILs, which are the main components of SASP secreted by senescent cells. Although the accumulation of senescent cells is a fundamental aging process, oxidative stress and TNF stimulation promote accelerated senescence of the RA-FLS, leading to its premature, excessive manifestation of Inflammaging, which promotes the release of inflammatory factors and contributes to the development of the pathological features typical of synovitis [41]. **(2) immunosenescence.** Senescent immune cells play an important role in inflammation-related diseases [42]. Inflammaging induces immunosenescence, and immunosenescence promotes Inflammaging, constituting a bidirectional positive feedback loop [43]. In this study, Plasma cells, CD8 T cells, memory-activated CD4 T cells, and follicular helper T cells were positively correlated with MMP9, CXCL10, and IL15 and negatively correlated with FOXO3, and these immune cells were aggregated in the synovial membrane of RA. The accumulating memory T cells are an important manifestation of inflammatory diseases in older adults [44]. Senescent T cells, the risk determinant of RA, can differentiate into pro-inflammatory phenotypes to accelerate the disease process [45,46], and the immune system in RA accelerates aging [47]. Immunosenescence and RA promote each other in a vicious circle.

### 4.3 The role of autophagy in aging and RA

Autophagy is a quality control mechanism capable of maintaining cellular homeostasis, and its protective effect on the organism is negatively correlated with age. The expression of ULK1, BECN1, and LC3 is down-regulated in senescent cells, and the age-induced decrease in autophagy levels contributes to the imbalance in cellular homeostasis [48]. The mTORC1 pathway inhibits autophagy. Sustained mTORC1 signaling is required to maintain senescence, which provides senescent cells with the required free amino acids to secrete SASP [49]. Maintaining autophagy and autophagic flux can resist the damage caused by SASP to cells and tissues [50]. In RA, inhibiting the PI3K/AKT/mTOR axis can enhance autophagy and suppress the pro-inflammatory response of FLS [51]. Mitophagy reduces ROS levels in RA by maintaining the stable functioning of the antioxidant system [52]. Thus, autophagy plays an important role in both aging and RA.

### 4.4 Inflammatory factors in RA and aging

Inflammatory cells in RA can secret large amounts of the same inflammatory factors as SASP (MMPs, CXCLs, and ILs), leading to tissue remodeling and chronic sterile inflammation [53]. MMP9, CXCL10, and IL15 were included in our RA disease model.

### 4.5 MMP9

MMPs are a family of enzymes responsible for matrix degradation and play an important role in the mechanisms of bone and joint injury, and they are significantly highly expressed in the synovial fluid of RA patients [54]. Itoh et al. found that MMP9 KO mice exhibited milder symptoms of RA than controls, and the results suggest that MMP9 is a key protein responsible for RA [55]. High levels of MMP9 in the synovial fluid of RA are closely related to the destruction of RA articular cartilage [56]. MMP9 is a major member of matrix metalloproteinase in SASP [57], and it is often used as a SASP marker for aging-related studies [58]. It has been shown that activation of the p38-MAPK/MMP9 pathway may be associated with impaired autophagy [59].

### 4.6 CXCL10

Chemokines promote the development of RA. CXCL10 can be used as a marker of disease activity in the blood of early RA patients, and a decrease in the serum concentration of CXCL10 often accompanies improvement in the clinical manifestations of RA patients [60]. Significantly high expression of CXCL10 can be detected in the synovial lining regions of RA [61]. Therefore, chemokines are considered promising therapeutic targets for RA [62]. As a recently discovered SASP factor, CXCL10 is secreted at high levels by senescent cells, and it participates in the inflammatory pathological process of aging as an important member of the pro-inflammatory SASP [63,64]. High expression of CXCL10 reduces autolysosome formation and blocks autophagic flux [65]. High levels of CXCL10 can be detected in autophagy-deficient cells [66].

### 4.7 IL15

RA-FLS can overly express IL15 [67], and high levels of IL15 are present in the synovial fluid and synovial membrane lining layer of RA, which has potent chemotactic and activating effects on T cells [68]. IL15 can induce TNF-α production by synovial T cells, further aggravating RA [69]. Therefore, IL15 is involved in the pathogenesis of RA, and selective targeting of IL15Rα is a relatively new therapeutic strategy for RA [70]. Proteomic studies have shown that IL-15 as an SASP factor not only reflects biological aging status [71] but also can be used to predict adverse events in terms of age and medical risk [72]. Up-regulation of IL15 expression, down-regulation of LC3A/B, and up-regulation of p62 can be detected in allergic asthmatic mice, and IL15 may be associated with impaired autophagic flux [73]. In summary, MMP9, CXCL10, and IL15 may be important biomarkers for RA progression, aging exacerbation, and autophagy impairment.

### 4.8 FOXO3

**Regulation of FOXO (1) Phosphorylation.** In this study, the MAPK+PI3K pathway positively regulated MMP9, CXCL10, and IL15, whereas it negatively regulated FOXO3. The PI3K/AKT pathway is a negative regulatory pathway of FOXO, which phosphorylates FOXO and prevents its nuclear localization from reducing its transcriptional regulatory function [74]. Moreover, the MAPK signaling pathway also phosphorylates FOXO3 [75]. **(2) Ubiquitination.** MDM2 with E3 ligase activity can bind to FOXO3, leading to its ubiquitination and degradation [76]. **(3) Acetylation.** Acetylation of proteins can hinder autophagy from proceeding, and the deacetylase Sirtuin-1 (SIRT1) extends lifespan by promoting autophagy [77]. Sirtuins are NAD(+)-dependent deacetylase, and SIRT1 can deacetylate FOXO3 to counteract oxidative stress and aging [78]. The NAD(+)/SIRT pathway can counteract aging by activating the FOXO signaling pathway, so drugs that enhance deacetylase activity have a potential role in aging diseases such as arthritis and osteoporosis [79]. **(4) Methylation.** The methyltransferase Set9 can methylate FOXO3 to increase its transcriptional activity [80]. To summarize, post-translational modification of FOXO acts as “The FOXO code”, which can selectively read proteins to regulate gene expression rapidly. We can control FOXO activity to prevent and treat aging and age-related diseases [81].

### 4.9 FOXO3 links to RA, aging, and autophagy

Up-regulation of FOXO3 levels prevents the proliferation of RA-FLS and the release of pro-inflammatory factors [82]. Viatte et al. reported that FOXO3 deficiency in RA mice will exacerbate the symptoms of arthritis and the histologic changes and that high expression of FOXO3 can limit the progression of RA [83]. The FOXO family is considered to be a signaling integrator for homeostasis maintenance. Increasing FOXO activity can extend life span. FOXO3 is one of only two genes that are closely related to aging/longevity [16]. The expression of FOXO3 is significantly negatively correlated with aging, especially in the skeletal muscle system. FOXO3 plays a key role in protecting the skeletal muscle system of primates from aging [84]. FOXO is a key factor in autophagy and lysosomal biogenesis, which regulates the transcription of various autophagy-related genes (e.g., ULK, BECN1, ATG5, ATG12) [85]. In addition, FOXO3 can inhibit mTOR-related regulatory proteins from reducing the activity of mTORC1, thus promoting autophagy [86]. Parkin is an E3 ubiquitin ligase that has multiple functions in mitochondrial homeostasis and cell signaling pathways. The regulatory role of Parkin in autophagy not only promotes cell survival, but also regulates excessive inflammation and oxidative stress. FOXO3 can upregulate Parkin expression at the transcriptional level. FOXO3 activation helps maintain the autophagy state of cells to regulate cell homeostasis [87]. This study further explored the FOXO3-Parkin signaling pathway in the context of RA. The results showed that downregulation of FOXO3 leads to disruption of the FOXO3-Parkin signaling pathway, downregulation of Parkin levels and reduced expression of LC3B, resulting in impaired autophagy. Expression of FOXO3 in the mouse skeletal muscle system is accompanied by an increase in a marker of autophagosome formation (LC3-GFP), whereas the knockdown of FOXO3 results in reduced autophagosome levels in the adult skeletal muscle system [88]. FOXO3 is at the hub of the cellular homeostatic feedback loop and can monitor autophagy to correct the state of autophagic flux [89]. Thus, FOXO3 might be a key biomarker for RA, aging, and autophagy.

### 4.10 Taurine

Eliminating senescent cells will alleviate aging-related diseases and prolong life [90]. FOXO3 is a protective gene that delays aging and reduces the risk of disease, and the use of compounds related to the activation of FOXO3 is one of the most direct therapeutic approaches to aging and RA [91]. Taurine has the following actions. **(1) Taurine treats RA.** Tau can reduce ROS levels and thus attenuate oxidative stress damage [92]. Taurine can be converted into taurine chloramine (TauCl) and taurine bromamine (TauBr), which have anti-inflammatory and cytoprotective effects. Both TauCl and TauBr can inhibit RA-FLS, which has pro-inflammatory properties [93]. TauCl can normalize the pathogenic functions of RA-FLS by inhibiting pro-inflammatory factors such as MMPs [94]. However, impaired TauCl production has been found in the synovial fluid of RA patients [95]. **(2) Taurine treats aging.** Depletion of taurine can be found in senescent animals. Taurine transporter knockout (TauT-KO) promotes cellular senescence and shortened lifespan and accelerates the appearance of skeletal muscle senescence and pathological features in mice [96]. Taurine is important in maintaining cellular redox homeostasis and cellular health, and it is a promising anti-aging drug [97]. **(3) Taurine promotes autophagy.** Taurine can exert hepatoprotective effects in rat hepatocytes by activating autophagy and attenuating the blockage of autophagic flux [98]. Taurine can also induce Beclin1 upregulation in nasopharyngeal carcinoma cells to activate autophagy and play an antitumor role [99]. **(4) Taurine promotes FOXO3 expression.** There are very few studies related to taurine and FOXO3. It has been shown that TUG1 (taurine upregulated gene 1) can competitively bind microRNA-9 and promote FOXO3 expression [100]. Another study showed that hypotaurine is a precursor of taurine, and the stress response-related transcription factors DAF-16/FOXO contribute to the beneficial longevity conferred by hypotaurine to Caenorhabditis elegans [101]. However, there is no relevant research evidence to suggest that the combination of taurine with FOXO3 will have any beneficial effect on disease or longevity, in which case it is important to carry out theoretical binding compatibility studies of taurine with FOXO3. This study is the first to simulate the binding mode between taurine and FOXO3 by molecular docking and molecular dynamics simulation to predict the structural affinity and stability of the co-crystals and to provide a structural basis for further investigation of the pharmacological mechanism. Finally, we indicated in in vitro experiments that taurine can increase FOXO3 expression, and further enhance the transcriptional regulatory effect of FOXO3 on Parkin through the FOXO3-Parkin signaling pathway, promoting the increased expression of the autophagy marker LC3B. This improves the autophagy function of cells in RA, reduces the accumulation of damaging substances in cells, and provides new strategies and evidence for the treatment of RA. In addition, future research needs to further explore the effects of taurine on MMP9, CXCL10 and IL15. However, this study has some limitations. Although the results of the theoretical research and in vitro validation data of this study are consistent with relevant studies in the existing literature, which further supports the role of taurine in the treatment of RA through FOXO3. However, these findings still need to be verified by further in vitro and in vivo experiments, especially in different types of cells and animal models, in order to more comprehensively evaluate the clinical application potential of this mechanism. Follow-up studies should continue to explore this mechanism in depth to ensure the reliability of the results.

## 5. Conclusion

We identified MMP9, CXCL10, IL15, and FOXO3 as important biomarkers for RA, cellular senescence, and autophagy using multiple bioinformatics analyses and machine learning. Down-regulation of FOXO3 expression may be an important manifestation of impaired/inhibited autophagy and cellular senescence, leading to the promotion of RA. This study provides theoretical analysis and self-evidence of the potential of taurine and FOXO3, and verifies it with in vitro experiments, which provides reliable evidence that taurine regulates autophagy and cellular senescence through FOXO3 to treat RA. More in-depth experimental research, including in vivo verification, is still needed in the later stages to comprehensively evaluate its clinical feasibility.

**Key-points**

- FOXO3 is an key biomarker of cellular senescence and autophagy mechanisms involved in rheumatoid arthritis.
- Downregulation of FOXO3 expression is an important signal that impaired/inhibited autophagy and cellular senescence promote rheumatoid arthritis.
- Taurine may serve as a promising therapeutic drug for rheumatoid arthritis by targeting FOXO3.

## Supporting information

S1 Data The analytical data and experimental raw data of this study.
(zip)

## Abbreviations

RA: Rheumatoid arthritis
DEGs: Differentially expressed genes;
HC: Healthy controls;
GO: Gene ontology;
KEGG: Kyoto encyclopedia of genes and genomes;
PPI: Protein-protein interaction;
SVM-RFE: Support vector machine-recursive feature elimination;
ANN: Artificial neural network;
LASSO: Least absolute shrinkage and selection operator;
WGCNA: Weighted gene co-expression network analysis;
GSEA: Gene set enrichment analysis;
ROS: Reactive oxygen species;
TF: Transcription factor;
ROC: Receiver operating characteristic curve;
AUC: Area under the curve;
FLS: Fibroblast-like synoviocytes;
MM: Module membership;
GS: Gene significance.
