# Supplementary figures and images for: Taurine is a potential therapy for rheumatoid arthritis via targeting FOXO3 through cellular senescence and autophagy
Source: PLoS One. 2025 Apr 16;20(4):e0318311. doi: 10.1371/journal.pone.0318311 (PMC12002484; doi:10.1371/journal.pone.0318311)

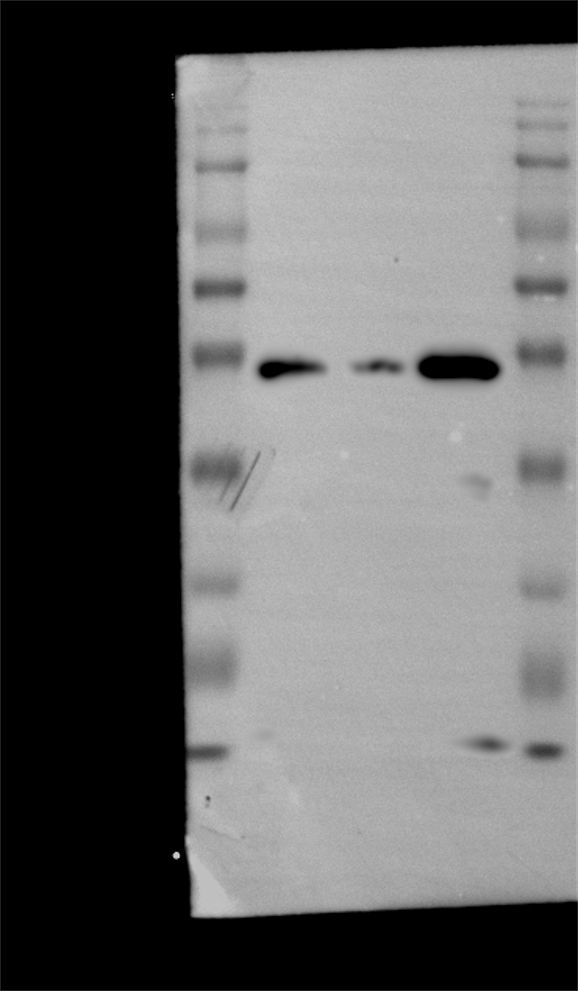

Supplement: S1 Data — The analytical data and experimental raw data of this study. (zip) [file pone.0318311.s001.zip › WB/Parkin.tif]

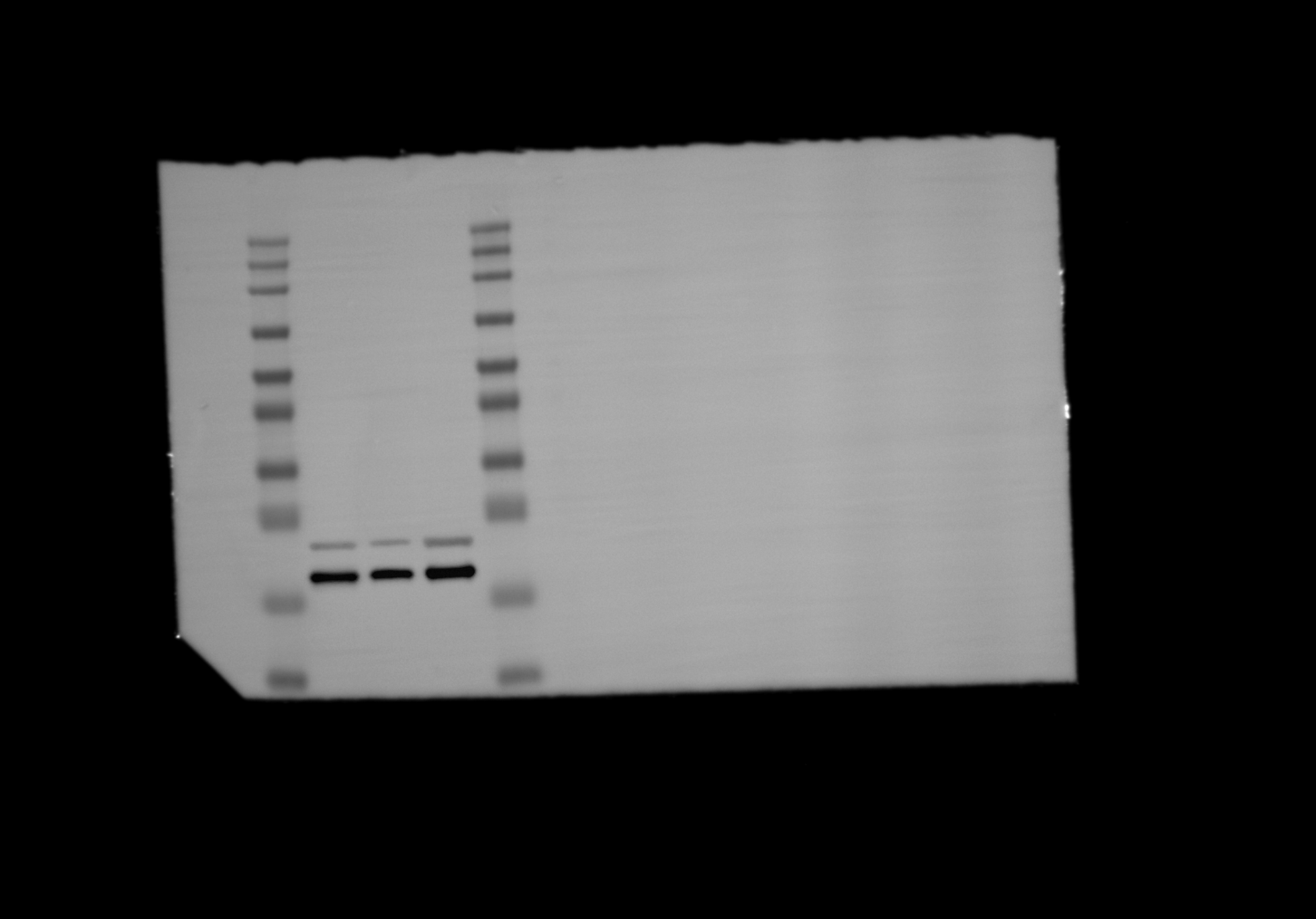

Supplement: S1 Data — The analytical data and experimental raw data of this study. (zip) [file pone.0318311.s001.zip › WB/LC3B.tiff]

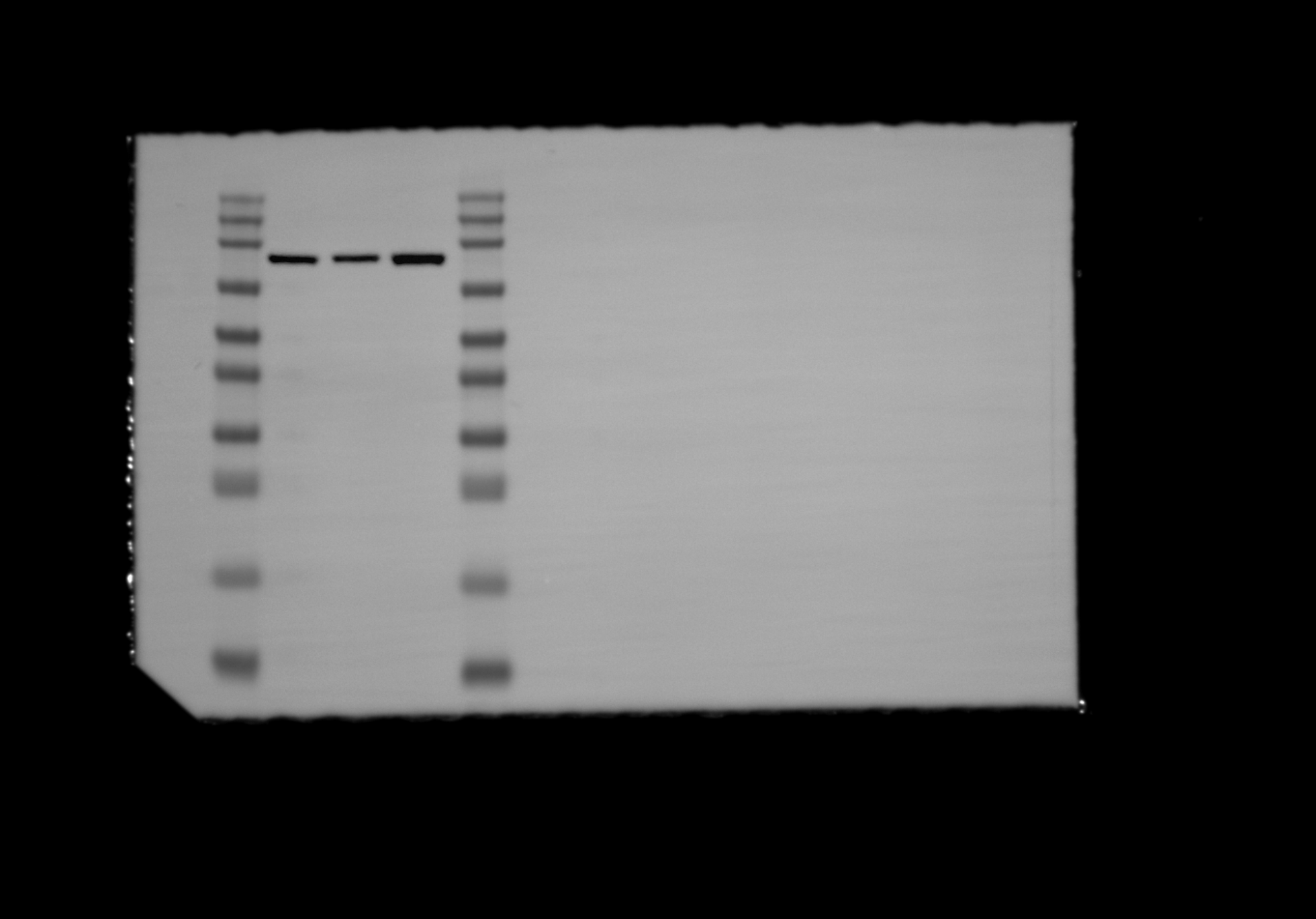

Supplement: S1 Data — The analytical data and experimental raw data of this study. (zip) [file pone.0318311.s001.zip › WB/FOXO3.tiff]

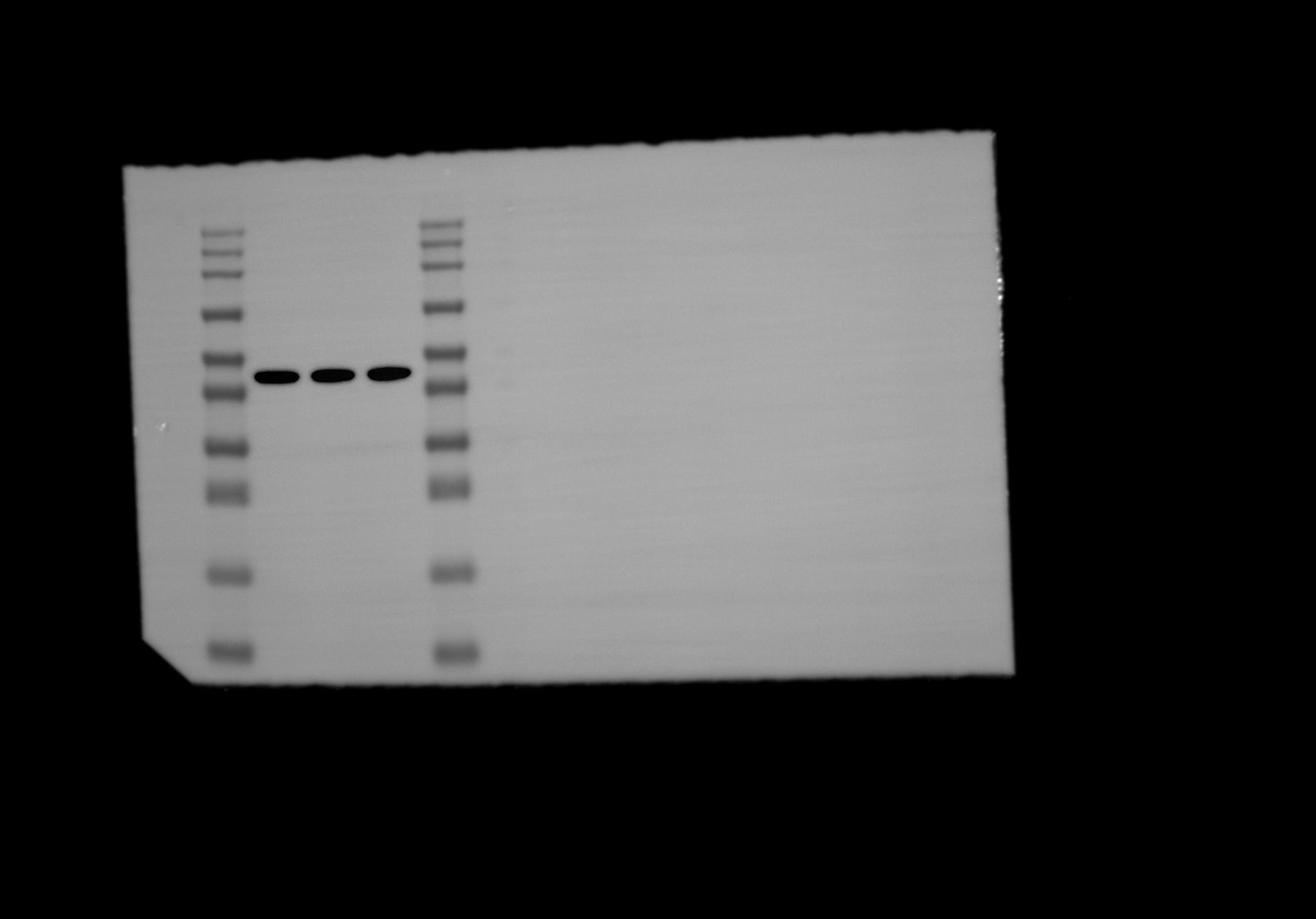

Supplement: S1 Data — The analytical data and experimental raw data of this study. (zip) [file pone.0318311.s001.zip › WB/β-actin.tiff]
